# Consensus clustering applied to multi-omics disease subtyping

**DOI:** 10.1186/s12859-021-04279-1

**Published:** 2021-07-06

**Authors:** Galadriel Brière, Élodie Darbo, Patricia Thébault, Raluca Uricaru

**Affiliations:** 1 CNRS, Bordeaux INP, LaBRI, UMR 5800, Univ. Bordeaux, 33400 Talence, France; 2 INRA, Bordeaux INP, NutriNeuro, UMR 1286, Univ. Bordeaux, 33000 Bordeaux, France; 3 INSERM U1218, Institut Bergonié, Univ. Bordeaux, 33076 Bordeaux, France

**Keywords:** Disease subtyping, Multi-omic data, Data integration, Consensus clustering

## Abstract

**Background:**

Facing the diversity of omics data and the difficulty of selecting one result over all those produced by several methods, consensus strategies have the potential to reconcile multiple inputs and to produce robust results.

**Results:**

Here, we introduce ClustOmics, a generic consensus clustering tool that we use in the context of cancer subtyping. ClustOmics relies on a non-relational graph database, which allows for the simultaneous integration of both multiple omics data and results from various clustering methods. This new tool conciliates input clusterings, regardless of their origin, their number, their size or their shape. ClustOmics implements an intuitive and flexible strategy, based upon the idea of *evidence accumulation clustering*. ClustOmics computes co-occurrences of pairs of samples in input clusters and uses this score as a similarity measure to reorganize data into consensus clusters.

**Conclusion:**

We applied ClustOmics to multi-omics disease subtyping on real TCGA cancer data from ten different cancer types. We showed that ClustOmics is robust to heterogeneous qualities of input partitions, smoothing and reconciling preliminary predictions into high-quality consensus clusters, both from a computational and a biological point of view. The comparison to a state-of-the-art consensus-based integration tool, COCA, further corroborated this statement. However, the main interest of ClustOmics is not to compete with other tools, but rather to make profit from their various predictions when no gold-standard metric is available to assess their significance.

**Availability:**

The ClustOmics source code, released under MIT license, and the results obtained on TCGA cancer data are available on GitHub: https://github.com/galadrielbriere/ClustOmics.

**Supplementary Information:**

The online version contains supplementary material available at 10.1186/s12859-021-04279-1.

## Background

Recent advances in biological data acquisition have made it possible to measure a wide range of data. Polymorphism data, DNA methylation, RNA expression, and copy number variations as well as other “omics” data are now routinely observed and analyzed. Each omics type has the potential to reveal different molecular mechanisms associated with a phenotype, and making use of all available omics data could decipher complex and multilevel molecular interactions. Though several integrative tools have been developed, with all of them aiming to answer biological questions by using multiple available data sources, the issue of omics data integration is far from solved. Along with the issue of omics data heterogeneity and integration, scientists are challenged with the diversity of strategies and methods available to answer the same biological question, each approach having its own perks and benefits.

The question of cancer subtyping is particularly representative of this kind of issue. By performing a clustering analysis, disease subtyping aims at detecting subgroups of patients (samples) showing similar characteristics. Even in the single-omics context, such analysis can be challenging, and numerous clustering strategies have been implemented and/or tested to this end: hierarchical clustering strategies, density-, distribution- or centroid-based strategies, supervised and unsupervised strategies, etc. The selection of a clustering method as well as of the optimal parameters to use is generally tricky. Moreover, the various biological mechanisms that are involved may vary from one patient to another: each tumor is different and has its own characteristics, both in the tumor cells themselves and in their interaction with their environment. As these mechanisms are not restricted to a single molecular level, the detection of groups of patients showing similar characteristics across different omics is a key issue to enable personalized medicine, which aims to offer patients a treatment adapted to the characteristics of their tumors.

This detection of groups of patients showing similar characteristics across different omics motivated the development of new computational methods implementing different strategies to analyze several omics datasets simultaneously (for detailed reviews, see [1, 2]). According to the classification proposed in [1], the *early integration* strategy consists of concatenating omics datasets in a large matrix and applying a clustering method conceived for single-omics data [3, 4]. However, *late integration* approaches first cluster each omics dataset independently and fuse single-omics clusterings into one multi-omics clustering [5, 6]. Other approaches perform *intermediate integration*, fusing sample similarities across omics [7–9], using dimension reduction strategies [10, 11], or statistical modeling with Bayesian frameworks [12–14].

To tackle both issues mentioned above, i.e., multi-omics and multi-strategy integration, one may want to apply a particular type of late integration strategy by taking multiple clustering results (using different data, methods and parameters) and fusing all of them into one *consensus clustering*. Such a consensus clustering should benefit from the complementary information carried by various omics data and capitalize upon the strengths of each method while fading their weaknesses.

Note that with respect to classical late integration strategies that start from the raw omics datasets (e.g., PINS [6] uses perturbations of raw omics data to generate the most stable multi-omics clustering), consensus clustering methods rely solely on clustering results. This property is essential, as it allows for any clustering algorithm and any clustering result to be used, regardless of the availability of the raw omics dataset and of its type.

A naive way to compute a consensus clustering would be to perform the *intersection* of the clustering results, i.e., by simply taking the associations on which all methods agree. However, the greater the number of clusterings to fuse, the smaller the intersection is. Moreover, when clusterings show different numbers of clusters, the question of the intersection is not trivial. Therefore, the issue of consensus clustering requires further methodological developments.

To compute a consensus clustering from a set of input clusterings, two main strategies exist: object co-occurrence-based approaches and median partition-based approaches [15]. In the former strategy, consensus clustering is computed from a matrix counting co-occurrences of objects in the same clusters [5]. The latter strategy focuses on finding a consensus clustering maximizing the similarity with the input partitions. Both strategies raise several nontrivial questions. The choice of a clustering algorithm and its tuning is not straightforward when working with a co-occurrence matrix. However, for the median partition-based approach, the choice of a similarity measure is determinant. Nevertheless, for consensus clustering in a multi-omics multi-method context, comparing co-occurrences of objects is more pertinent than comparing similarities between partitions.

Here, we present ClustOmics, a new graph-based multi-method and multi-source consensus clustering strategy. ClustOmics can be used to fuse multiple input clustering results, obtained with existing clustering methods that were applied on diverse omics datasets, into one *consensus clustering*, regardless of the number of input clusters, the number of individuals clustered, the omics and the methods used to generate the input clusterings.

The co-occurrence strategy implemented in ClustOmics (detailed in the “Methods” section) is based on *evidence accumulation clustering* (EAC), first introduced by Fred and Jain [16]. The idea is to consider each partition as independent evidence of data organization and to combine them using a voting mechanism. Similar to clustering methods that use a distance or a similarity measure to compare objects, EAC considers the co-occurrences of pairs of objects in the same cluster as a vote for their association. The underlying assumption is that objects belonging to a *natural* cluster are more likely to be partitioned in the same groups for different data partitions. Thus, one can use the counting of the co-occurrences of the objects in clusters as a pairwise similarity measure. We further refer to these co-occurrence counts as the *number of supports*. This measure, summarizing the results from the input clusterings, is a good indicator of the agreement between the partitions and allows production of a new partitioning that can be qualified as consensual. Although computationally expensive, this strategy allows exploiting all clustering results, regardless of the number of clusters and their size and shape.

We designed ClustOmics as an exploratory tool to investigate clustering results in order to increase the robustness of predictions, taking advantage of accumulating evidence. To allow the user to tackle a specific question and to explore relationship patterns within input clusterings and generated consensus, we store the data in a non-relational graph-based database implemented with the Neo4j graph platform [17]. The use of a graph native database facilitates the storage, query and visualization of heterogeneous data, hence allowing the development of a solution that is flexible to various integration strategies. Indeed, by fusing clusterings from different clustering methods, different data types, different experimental conditions, or several options at the same time, through the use of what we call *integration scenarios*, ClustOmics can address a wide range of biological questions.

ClustOmics was applied in the context of multi-omics cancer subtyping, with TCGA data from different cancer types and multiple omics datasets. Input clusterings were computed with several single and multi-omics clustering methods and then were fused in a consensus clustering. Further details on the strategy implemented in ClustOmics are given in “Methods” section. To assess the benefit of this novel method, we further explored the robustness of our consensus clusterings with respect to the input clusterings, as well as their biological relevance, based on clinical and survival metadata available for each patient. We compare the ClustOmics results with those of COCA [5], a well-known co-occurrence-based consensus clustering tool that has already been used to combine multiple omics datasets to reveal cancer subtypes [18].

## Results

Consensus clustering for disease subtyping in a multi-omics context can be implemented as an a priori solution making a consensus of omics-specific input clusterings or by a posteriori computing a consensus from multi-omics input clusterings. To better understand the perks and benefits of fusing omics data in one way or another, ClustOmics was tested in these two contexts based on two *integration scenarios*.

**Fig. 1:**
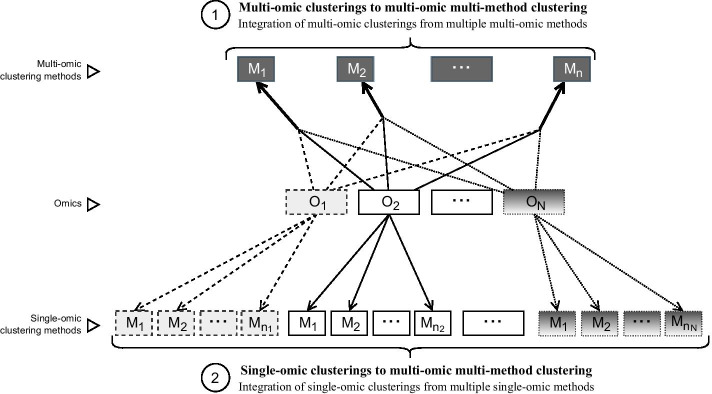
Two integration scenarios: multi-to-multi consensus clustering and single-to-multi consensus clustering. Arrows are dashed according to the omics considered by each input clustering method

First, we used ClustOmics to fuse multi-omics clusterings computed with existing integrative methods. In this scenario (multi-to-multi, MtoM), the integration of omics is performed by various existing clustering tools, and ClustOmics computes a consensus result of the different multi-omics clusterings produced. The second scenario (single-to-multi, StoM) involves both methods and omics integration, as only single-omics clusterings computed from various methods are fused into one consensus clustering. See Fig. 1 for a visual representation of these two scenarios.

Below, we analyze and compare ClustOmics and COCA consensus multi-omics clusterings (produced using the same set of input clusterings) for the two integration scenarios, on TCGA data from ten different cancer types, three omics datatypes (gene expression, miRNA expression and methylation) and various input clustering strategies (described in “Methods - Datasets and tools used for computing input clusterings” section). We further focus on breast cancer and analyze the ClustOmics results for the single-to-multi scenario on the breast dataset.

### Results overview of the ten cancer types for the two integration scenarios

Running ClustOmics and COCA on the ten cancer datasets with respect to the different integration scenarios implies starting by computing single- and multi-omics input clusterings to group patients according to their single- and multi-omics profiles.

For the multi-to-multi (MtoM) scenario, multi-omics input clusterings were obtained with existing multi-omics clustering tools: PINS [6], SNF [7], NEMO [8], rMKL [9] and MultiCCA [10] (see Table 6).

For the single-to-multi (StoM) scenario, the same tools listed above were applied, except for the MultiCCA tool, which can only be used in a multi-omics context and was replaced with the simple yet robust state-of-the-art method, K-means clustering [19]. In this scenario, the tools were applied to each omics dataset independently. Moreover, to evaluate the benefits of including patients with missing data (that were not measured for all of the three omics), two different runs were performed. In the first run, referred to as *StoM OnlyMulti*, only patients measured for the three omics were considered, that is, patients with no missing data. For the second run, named *StoM All*, all available patients for each omics were kept, implying that in this scenario the set of patients clustered in input clusterings was different across omics.

**Fig. 2:**
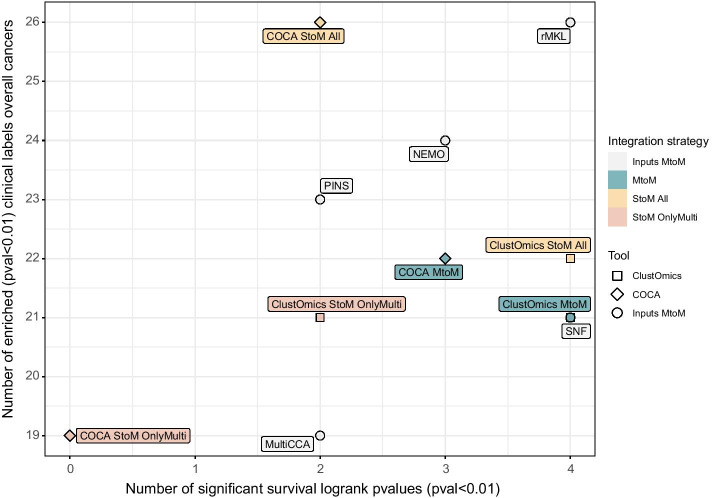
Overview of survival and clinical label enrichment results for the ten cancer types analyzed. The *x*-axis represents the number of significant survival *p* values ($< 0.01$) found for each clustering, over all the ten cancer types. The *y*-axis represents the total number of significantly enriched clinical labels (*p* values $< 0.01$), all cancer types included. In total, 79 enrichment *p* values were computed from 32 distinct clinical labels

A survival and clinical label enrichment analysis was conducted on ClustOmics and COCA multi-omics consensus clusterings, as well as on the single-omics and multi-omics input clusterings (see “Methods - Biological metrics” section for more details on the biological metrics used). An overview of the results for the ten cancer types is displayed in Fig. 2.

In terms of clinical label enrichment in clusters, the number of clinical labels significantly enriched varies from 19 to 26 (for a total of 79 enrichment *p* values computed from 32 distinct clinical labels), depending on the clustering tool. The majority of clinical labels found enriched in ClustOmics consensus clusters were also found enriched for at least one input clustering as well as in the corresponding COCA consensus, and clinical labels stably enriched in input clusterings were also found enriched in ClustOmics and COCA consensus clusterings. For details with respect to the distribution of the clinical labels found enriched in input and consensus clusterings for the MtoM and StoM scenarios, see Additional file 1: Figure S1.

The survival analysis results show high heterogeneity, supporting the idea of computing a consensus clustering, especially when no gold-standard metric or ground-truth data are available. In this sense, it is important to stress out that ClustOmics succeeded to compute biologically relevant consensus partitions from input clusterings of variable quality. Indeed, in the multi-to-multi case, ClustOmics managed to find 4 out of 10 significant log-rank *p* values, counterbalancing the PINS and MultiCCA mitigated results, although they were part of the input clustering results used for this integration scenario. For the same set of input clusterings, COCA MtoM yielded to a 3 survival-wise significant consensus result.

Interestingly, for the single-to-multi scenario, Fig. 2 clearly shows that considering individuals with missing data (StoM All) greatly improves the consensus clusterings, both in terms of clinical label enrichment and survival analysis, for ClustOmics as well as for COCA. For the StoM All scenario, COCA found 4 additional enriched clinical labels with respect to ClustOmics but yielded only 2 out of 10 survival-wise quality clusterings, compared to 4 for ClustOmics. The quality results for omics-specific input clusterings used for this scenario do not appear in Fig. 2 but are further detailed in “Results - Integration of single-omics clusterings” section.

Detailed results for the MtoM and StoM integration scenarios are given in the following two sections.

### Integration of multi-omics clusterings (multi-to-multi scenario)

Input multi-omics clusterings were computed with the five multi-omics clustering methods presented in “Methods - Datasets and tools used for computing input clusterings” section using default parameters and following recommendations of the authors. The clusterings were produced using the multi-omics patients exclusively (those for which all three omics data are available). To make all input clusterings comparable, we ran NEMO in the same way, though compared to the other five tools, NEMO is able to handle partial data.

ClustOmics was run with the *min_size_cluster* parameter arbitrarily set to 8 nodes for all cancer types, meaning that clusters of size below 8 were removed from the consensus clustering, with the corresponding individuals being reassigned to consensus clusters exceeding the size threshold. We also set the *min_size_consensus* parameter to $95\%$ of the population to ensure that less than $5\%$ of individuals are being reassigned to consensus clusters, either because of the number of support thresholds on the integration graph or because of the filter on the size of the clusters. The quantitative global measures on the ClustOmics consensus clusterings are detailed in Table 1.

**Table 1:** Multi-to-Multi Scenario: Number of patients initially clustered by ClustOmics, number of patients reassigned to consensus clusters, number of supports used to filter the integration graph, number of consensus clusters generated by ClustOmics and COCA, and average number of clusters in input clusterings

Cancer	# patients clustered	# patients reassigned	# supports threshold	# clusters ClustOmics	# clusters COCA	Avg # clusters inputs
AML	165	5	4	6	3	4.6
BIC	619	2	4	5	2	4.0
COAD	220	0	2	3	3	5.2
GBM	267	7	4	3	3	3.4
KIRC	176	7	3	3	3	4.2
LIHC	363	4	4	5	2	3.2
LUSC	341	0	2	2	2	3.4
OV	285	2	4	5	2	3.4
SARC	257	0	3	3	3	3.4
SKCM	351	0	3	5	3	4.8

Note that the maximum number of supports promoting the association of two patients in the same consensus cluster is bounded to 5, as five input clusterings (computed from five integrative clustering tools) were used for this integration scenario. After testing all possible thresholds on the number of supports, the optimal filtering threshold was obtained for each cancer type (2, 3 or 4, depending on the cancer type), meaning that only pairs of patients clustered in the same multi-omics cluster by at least 2 to 4 clustering methods were considered to compute the consensus clustering.

When comparing input and ClustOmics consensus clusterings, we observe a certain consistency in terms of the number of clusters. COCA, however, resulted in 2 to 3 clusters independently from the cancer type, which suggests a lower sensitivity to input clustering dissimilarities compared to ClustOmics.

Two cancer datasets, COAD and LUSC, showed the lower consistency between the input predictions and clustered with a number of supports of 2. For LUSC cancer type, the consensus clustering resulted in only 2 clusters, despite the large size of the available cohort (341 individuals). The computation of the adjusted Rand index (ARI) [20] between input clusterings, a measure of similarity between partitions, showed that for these two cancer types, SNF and NEMO clusterings were very similar (with an ARI value of 0.7 for COAD SNF and NEMO clusterings and of 0.9 for LUSC; see Additional file 1: Figure S2) while the 3 other input clusterings showed high pairwise dissimilarity ($ARI \le 0.4$). The resulting consensus clusterings for both ClustOmics and COCA were very similar to SNF and NEMO and dissimilar to the other input clusterings, failing to compute an actual consensus of all input partitions. For the other cancer types, similarities between input clusterings were more balanced, enabling ClustOmics to reconcile predictions. ARI heatmaps comparing input and consensus clustering similarities for the ten cancer types in the MtoM scenario are available in Additional file 1: Figure S2.

Unsurprisingly, from Table 1, we remark that filtering the integration graph with a higher number of supports generally results in reassigning individuals (from 2 to 7) to consensus clusters. The predictions regarding these reassigned patients do not necessarily meet the number of supports threshold for which the consensus clusters were computed.

**Fig. 3:**
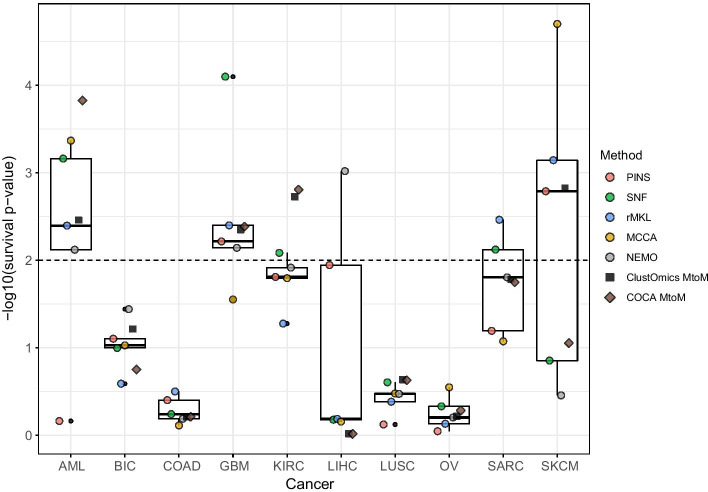
Survival analysis results for ClustOmics and COCA multi-to-multi consensus clustering and for each input multi-omics clustering. The horizontal dashed line indicates the threshold for significantly different survival rate (*p* value $\le 0.01$). Boxplots were computed considering input clusterings only

Figure 3 presents survival analysis results for the various multi-omics clusterings given as input to ClustOmics and COCA MtoM and for the resulting consensus clusterings. When looking at the input clustering survival results, we can differentiate two cases:

- For AML, LIHC, SARC and SKCM, the input clusterings show a quite high heterogeneity in terms of survival quality
- For BIC, COAD, GBM, KIRC, LUSC and OV cancer types, the input clusterings show relatively homogeneous survival quality

For the first group of cancer types, the heterogeneity of input clustering survival qualities indicates how the choice of one clustering method can drastically impact the results. For these cancer types, ClustOmics produced consensus clusterings of a survival-wise quality approaching the median quality value, considering the input clusterings. Indeed, from input clusterings of various quality, ClustOmics was able to extract the most stable patterns across the input partitions.

When input partitions show homogeneous survival quality, ClustOmics gives similar results, which is an expected behavior. The largest deviation from the median is found for the KIRC cancer type, for which both COCA and ClustOmics consensus clusterings produced a partition of higher quality than could have been expected.

Consensus clusterings were also investigated for clinical labels enriched in clusters. For the ten cancer types, ClustOmics and COCA found 20 common clinical labels as being enriched, of which 19 were also found enriched in at least one input clustering. However, 16 labels were found as enriched in at least one input clustering but not in the consensus clusterings (see Additional file 1: Figure S1B). Table 2 give complete details on the clinical labels enriched in ClustOmics consensus clusterings for the ten cancer types.

**Table 2:** Clinical labels found enriched in multi-to-multi (MtoM) scenario consensus clusters, in single-to-multi (StoM All) scenario consensus clusters, and for both scenarios

Cancer	Scenarios	Enriched clinical labels
AML	Both	Age at initial pathologic diagnosis
		CALGB cytogenetics risk category
		Leukemia French–American–”British Morphology Code
BIC	Both	Age at initial pathologic diagnosis, PAM50 call
		Pathologic N, Pathologic Stage, Histological type
		Estrogen receptor status, Progesterone receptor status
	StoM All	Pathologic M, Pathologic T
COAD	MtoM	Histological type
	StoM All	Age at initial pathologic diagnosis
GBM	Both	None
KIRC	Both	Pathologic M, Neoplasm histologic grade
	StoM All	Pathologic T
LIHC	Both	Gender, Age at initial pathologic diagnosis, Fetoprotein outcome value
LUSC	Both	None
OV	Both	None
SARC	Both	Gender, Age at initial pathologic diagnosis, Histological type
	MtoM	New neoplasm event type
SKCM	MtoM	Age at initial pathologic diagnosis

For AML, for example, ClustOmics computed clusters enriched for the CALGB cytogenetics risk category, a risk classification based on the Cancer and Leukemia Group B clinical trial [21], and for the French–American–British (FAB) morphology code, a clinical classification for AML tumors [22]. Reassuringly, BIC consensus clustering was found enriched for the PAM50 classification, a widely used breast-cancer subtype predictor [23].

### Integration of single-omics clusterings (single-to-multi scenario)

To assess ClustOmics performance when fusing simultaneously input clusterings computed from different omics data and with different clustering methods, we investigated a second integration scenario, combining single-omics clusterings produced independently on each omics dataset. The overall cancer consensus results for this scenario are displayed in Fig. 2 and discussed in detail in this section.

As stated above, single-omics clusterings were computed using the following five clustering tools: PINS [6], SNF [7], NEMO [8], rMKL [9] and K-means clustering [19] (with an optimal number of clusters computed with the Silhouette index [24]).

To assess the benefit of including individuals with missing data (not measured for all omics), two analysis were performed for this scenario:

- For StoM OnlyMulti, input clusterings were computed using exclusively multi-omics patients. Given that this is the same set of individuals as in the MtoM integration scenario, the same parameters were used for all cancer types, i.e., $min\_size\_consensus = 95\%$ and $min\_size\_cluster = 8$.
- For StoM All, omics clusterings were computed using all available patients. As the proportion of missing data varies between cancer types (up to $66\%$ partial data for KIRC; see Table 5) and between omics, input clusterings do not apply to the same set of patients as in the scenarios previously described. To account for this increase in the number of patients to be clustered, $min\_size\_cluster$ was set to $5\%$ of the *multi-omics* population. The $min\_size\_consensus$ parameter was set to $95\%$ of the *multi-omics* population.

As shown in Fig. 2, fully exploiting the available data (including patients with missing data) greatly improved the consensus clusterings, for both ClustOmics and COCA. Moreover, for 3 cancer types, BIC, GBM and LUSC, capitalizing on all available individuals resulted in increasing the number of supports used to filter the integration graph. The largest increase in the number of supports threshold was observed for BIC, i.e., from 7 supports in the StoM OnlyMulti up to 11 in the StoM All run. For LIHC, OV and SKCM, however, we observe a decrease of $-2$, $-1$ and $-1$, respectively, in the number of supports. For the other cancer types, the threshold on the number of supports is identical between the two runs. In the follow-up of this study, we will focus on the results of the StoM All run.

In this scenario, the maximum possible number of supports is 15 as the five clustering methods were run on three omics datasets for each cancer. Note that for this scenario, the threshold on the number of supports used to filter the integration graph has great influence on the capacity of ClustOmics to produce consensus clusters across omics and on the interpretation of the results. Indeed, the threshold has to be greater than 5 to ensure that all the conserved integration edges rely on an association that is consistent across at least two different omics (one omics type being represented by five input clusterings). To ensure that all integration edges are built upon all three omics, the threshold must be 11 or higher. One should estimate an acceptable threshold depending on the experimental design and the biological question to address.

In our case, as we did not wish to bring any a priori preconceptions on which omics should have a stronger impact on the results (indeed, one omics data type could particularly well explain the disparities in molecular profiles of patients for a cancer type but not for the others), we considered a number of supports of 7 to be sufficient to ensure that selected integration edges are either moderately consistent across the three omics or strongly consistent in one omics type.

Together with the constraint to preserve at least $95\%$ of the multi-omics population (*min_size_consensus* parameter), this gave a number of supports used to filter the integration graph ranging from 7 to 11. The results for this scenario are displayed in Table 3.

**Table 3:** Single-to-Multi All Scenario: Total population size (which are multi-omics), number of patients clustered or reassigned to consensus clusters (which are multi-omics), number of supports used to filter the graph, number of clusters generated by ClustOmics, number of clusters generated by COCA, and average number of clusters in the input clusterings

Cancer	Total (multi-omics)	Clustered (multi-omics)	Reassigned (multi-omics)	# supports threshold	# clusters ClustOmics	# clusters COCA	Avg # clusters inputs
AML	197 (170)	176 (163)	21 (7)	8	7	4	6.60
BIC	1096 (621)	600 (600)	496 (21)	11	6	2	3.87
COAD	303 (220)	276 (220)	27 (0)	7	6	4	3.47
GBM	578 (274)	434 (262)	144 (12)	9	11	2	4.13
KIRC	534 (183)	316 (174)	218 (9)	9	9	2	4.07
LIHC	377 (367)	363 (354)	14 (13)	8	5	2	4.73
LUSC	501 (341)	337 (321)	164 (20)	8	4	2	4.73
OV	591 (287)	393 (272)	198 (15)	9	9	2	3.47
SARC	261 (257)	261 (257)	0 (0)	8	3	3	4.33
SKCM	368 (351)	345 (329)	23 (22)	8	6	4	4.67

One of the major benefits of this integration scenario (in addition to the fact that single-omics clusterings are easier to compute) is its ability to cluster individuals that did not appear in all input partitions. Interestingly, although multi-omics patients have better chances to show high numbers of supports (as they appear in all input clusterings), some proportion of those multi-omics patients had to be reassigned to consensus clusters, while other individuals who were not measured for the 3 omics were clustered immediately, which suggests a good agreement between the input clusterings for the classification of these individuals.

Input clusterings can show great similarity for a given omics type. If this omics type allows differentiation of groups of individuals in a clear-cut way, it will drive consensus clustering. However, if the omics type is less relevant to partition patients, input clusterings are more likely to show different patient associations. Such clusterings add noise-like integration edges in the integration graph, with low number of supports on edges. Therefore, we expect each omics to have a different impact on the final consensus clustering. To evaluate the impact of omics-specific input clusterings on the consensus result, we used the adjusted Rand index (ARI) [20].

**Fig. 4:**
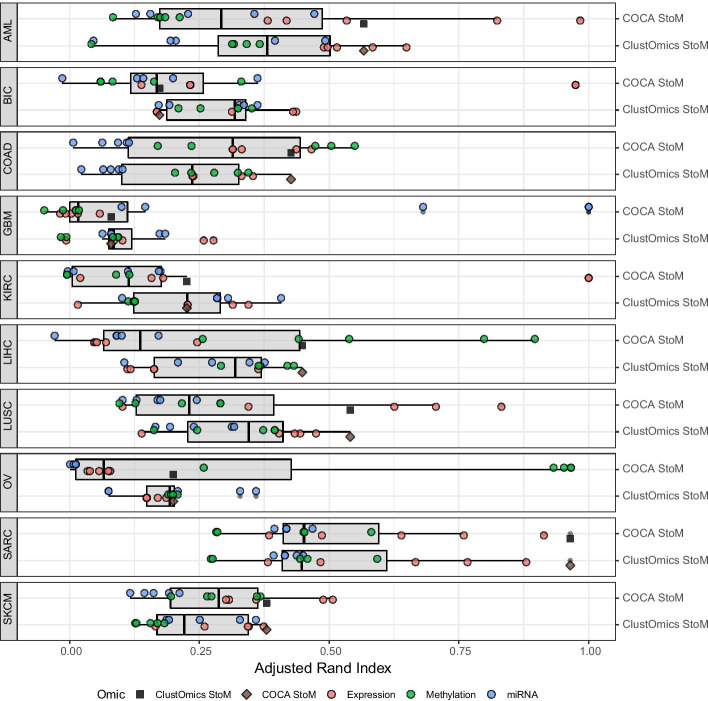
Adjusted Rand index of input clusterings relative to ClustOmics and COCA StoM consensus multi-omics clusterings. Each point corresponds to one clustering and is colored according to the omics type used. Each omics dataset was clustered using five different clustering tools (PINS, NEMO, SNF, rMKL, K-means), and therefore, it is represented by five input clusterings. ClustOmics and COCA respective consensus clustering similarity is displayed with a black square and a brown diamond

In Fig. 4, ClustOmics and COCA consensus clusterings were compared to each of the input clusterings. The relative proximity of a clustering consensus to the different input clusterings, as measured by the ARI, indicates the ability of the tool to produce a partition that can genuinely be considered as reconciling the input predictions. In that respect, the ARI of a consensus clustering in relation to its inputs should be maximized for a maximum of input clusterings, including for those coming from different omics. The highest similarity between consensus and input clusterings from different omics sources is observed for the SARC cancer dataset (see Fig. 4), with ARI values ranging from 0.4 up to 0.9 for at least one input clustering computed from each of the three omics datasets, suggesting similar associations at different molecular levels. For the other cancer types, the agreement between omics sources is less straightforward. Interestingly, for all cancer types, COCA and ClustOmics consensus clusterings resemble the same input clusterings (computed from the same set of omics sources), thus suggesting that some omics are more appropriate to explain molecular differences between individuals. Unsurprisingly, gene expression impacts consensus clustering on most cancer types, but miRNA and methylation data also guided consensus clusterings, especially in COAD, LIHC and OV. Figure 4 also shows that the dispersion of ARI values is much greater for COCA consensus clusterings than for ClustOmics. While COCA consensus clusterings are very similar (if not identical) to a few input clusterings but very dissimilar to the others, ClustOmics produces a consensus that is closer in average to all inputs.

**Fig. 5:**
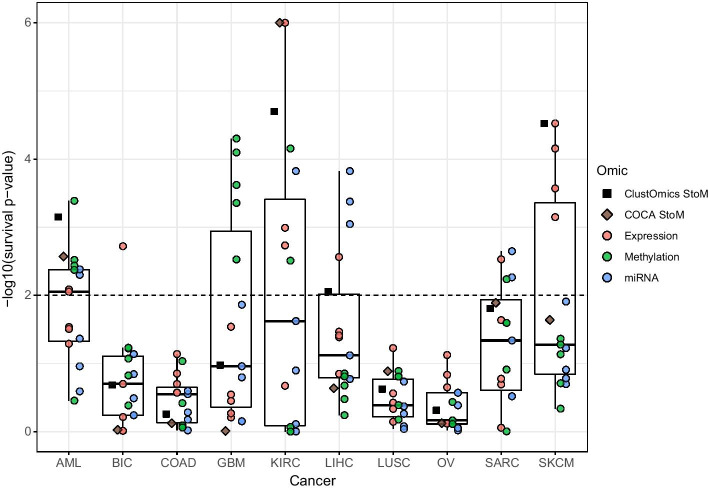
Survival analysis results for ClustOmics and COCA single-to-multi All consensus clustering and for each input multi-omics clustering. The horizontal dashed line indicates the threshold for significantly different survival rate (*p* value $\le 0.01$). Boxplots were computed considering only input clusterings

Survival analysis for this integration scenario (see Fig. 5) shows two groups of cancer types, as already noted for the multi-to-multi scenario. For BIC, COAD, LUSC and OV, gene expression, methylation and miRNA input clusterings show homogeneous survival *p* values. For these cancer types, ClustOmics computed a consensus clustering with similar quality scores. For the cancer datasets showing higher heterogeneity among input clusterings survival quality, ClustOmics found significant survival *p* values for AML, KIRC, LIHC and SKCM, despite some low-quality clusterings that were given as input.

Clinical labels found enriched in consensus clusters are listed in Table 2. AML clusters were found enriched for both CALGB and FAB classifications, KIRC clusters for histologic grade and pathologic M and T (referring to the TNM classification of tumors [25]), LIHC clusters for gender, age at diagnosis and fetoprotein outcome value, while SKCM clusters showed no enriched clinical parameters. While BIC consensus clustering did not show good survival-wise results, pathologic M, N and T labels were found enriched in clusters, as well as pathologic stage, histological type, PAM50 call, and estrogen and progesterone receptor status.

In the following section, we further explore the single-to-multi consensus clustering for BIC dataset.

### Study case: BIC single-to-multi consensus clustering

In this section, we focus on the consensus clustering of the 15 single-omics clusterings for the BIC dataset (five clustering methods, listed in the previous section, applied on three omics data types) and analyze these results in parallel to the PAM50 classification. As the PAM50 classification is computed from the expression of 50 specific genes, while in this work, we capitalize on three different omics, a certain heterogeneity in the clusters when compared to the PAM50 prediction is expected. Moreover, this heterogeneity is to be further explored, as it could reveal subtypes that are not distinguishable when considering only PAM50 genes but that are heterogeneous when integrating other data sources.

From the 1096 patients available in the BIC dataset (of which only 621 patients are measured for the three omics), ClustOmics succeeded in primarily classify 600 multi-omics patients in consensus clusters, with a number of supports threshold of 11 (see Table 3). The remaining 21 multi-omics individuals were reassigned to consensus clusters, as well as the 475 individuals with missing data. The consensus clustering resulted in a partition with 6 clusters, with sizes ranging from 115 to 254 individuals (the minimum allowed size for a cluster *min_size_cluster* being set to 5% of the multi-omics population, that is, 31 individuals for BIC).

As the PAM50 clinical labels were missing for 255 patients, we applied the original classifier introduced by Parker et al. [23] to call the missing labels. To estimate the quality of reassessed PAM50 labels, we evaluated the concordance between available PAM50 labels and recomputed PAM50 labels. The F1-scores showed the Basal, Luminal A, Luminal B and Her2 PAM50 labels to be well predicted (F1-score of 0.89, 0.75, 0.74 and 0.64, respectively). Predictions for the Normal-like class are less reliable (F1-score of 0.27) due to the small size of the class (23 individuals).

**Fig. 6:**
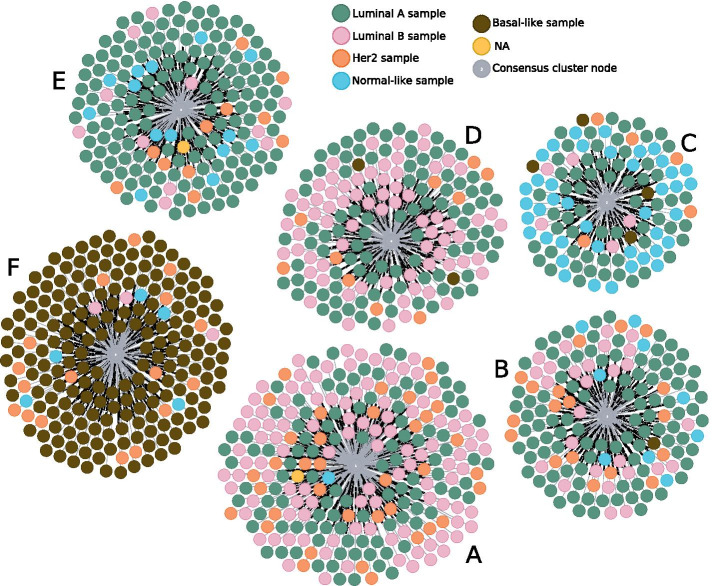
BIC consensus clustering with patients colored according to the PAM50 prediction. Annotated screenshot from the Neo4j browser for graph visualization

We further mapped the PAM50 calls to ClustOmics consensus clusters and observed significant concordance, as depicted in Fig. 6.

Indeed, Luminal A samples are overrepresented in the consensus clusters *B* and *E*, Luminal B samples in *A* and *D*, Her2 samples in *A*, and Normal-like samples in consensus cluster *C* (see Fig. 6 and Table 4). The vast majority of basal-like samples were classified in consensus cluster *F*, which gathers 190 of the 197 basal samples, with the remaining 7 being clustered in consensus clusters *B*, *C* and *D*.

This mapping of PAM50 calls on consensus clusters, which seems fuzzy at first glance, is not surprising as it has been shown that separation of Luminal A and B samples was not reconstructed by RNA-seq unsupervised analysis [26]. Several studies also reported that the separation between Luminal subtypes was not consistent, suggesting that Luminal A and Luminal B samples may represent part of a continuum rather than distinct subgroups [26–28].

Moreover, clinical label enrichment analysis and additional tests applied to describe the clusters show good mapping between ClustOmics clusters and key biological clinical labels such as estrogen receptor and progesterone receptor status (ER/PR) or histological type of tumor (see Table 4).

**Table 4:** Over- and underrepresented clinical labels within BIC consensus clusters. ER+/ER− and PR+/PR−, respectively, correspond to estrogen receptor status and progesterone receptor status, positive and negative. M, N, and T stages refer to the TNM staging system

Cluster	Over-represented labels	Under-represented labels
A	Infiltrating Ductal Carcinoma	Infiltrating Lobular Carcinoma
	Her2, Luminal B	Basal, Normal-like
	ER+	ER−
B	MX, N3, T3	M0
	Stage III	
	Infiltrating Lobular Carcinoma	Infiltrating Ductal Carcinoma
	Luminal A	Basal
	ER+, PR+	ER−, PR−
C	MX, T3	M0, T2
	Infiltrating Lobular Carcinoma	Infiltrating Ductal Carcinoma
	Normal-like	Basal, Luminal B
	ER+, PR+	ER−, PR−
D	Stage X	
	Mucinous Carcinoma	Infiltrating Lobular Carcinoma
	Luminal B	Basal, Normal-like
	ER+, PR+	ER−, PR−
E	T1	
	Stage I	
	Luminal A	Basal, Luminal B
	ER+, PR+	ER−, PR−
F	N0	
	Stage II	Stage III
	Infiltrating Ductal Carcinoma, Medullary Carcinoma, Metaplastic Carcinoma	Infiltrating Lobular Carcinoma
	Basal	Luminal A, Luminal B
	ER−, PR−	ER+, PR+

Finally, we investigated the biological relevance of ClustOmics consensus clustering by comparing gene expression profiles between clusters. We computed the top 1000 genes differentially expressed across groups by applying the Kruskal–Wallis test [29] and selecting FDR adjusted *p* values below 0.001 (see Additional file 1: Figure S3). We clustered the top 1000 genes in 6 clusters using hierarchical clustering and for each gene list, we looked for overrepresented biological process (BP)-related Gene Ontology terms (GO terms). One of the gene clusters showed no significant results (FDR adjusted *p* values $\ge 0.05$), but the other 5 gene lists were found enriched for cilium organization and assembly, response to transforming growth factor $\beta$, tissue migration, T-cells activation, mitotic nuclear division or other biological processes (see Additional file 1: Figure S4).

More precisely, we found that the gene cluster *X*2, associated with cilium organization and assembly, microtubule bundle formation and regulation of intracellular steroid hormone receptor signaling pathway, was downregulated in consensus cluster *F* (composed mainly of Basal-like samples), compared to other consensus clusters. Gene cluster *X*3 was associated with response to transforming growth factor $\beta$, extracellular organization, transmembrane receptor protein serine/threonine kinase signaling pathway and regulation of muscle cells. Those genes appear downregulated in consensus clusters *A* (Her2, Luminal B samples), *D* (Luminal B samples) and *F* (Basal-like samples). Gene cluster *X*4, associated with epithelium migration and astrocyte differentiation, was found downregulated in consensus clusters *A* and *D* (both enriched in Luminal B samples) and upregulated in consensus cluster *F* (Basal-like samples). Gene cluster *X*5 is associated with T-cell activation, lymphocyte and leukocyte differentiation, membrane raft organization and regulation of peptidase activity and is downregulated in consensus clusters *A* and *D* (Luminal B samples). Finally, gene cluster *X*6, related to chromosome segregation and mitotic nuclear division, was found upregulated in consensus cluster *F* (Basal-like samples) and downregulated in consensus clusters *B* (Luminal A), *C* (Normal-like) and *E* (Luminal A).

## Discussion

The novel method that we present in this paper deals with two key issues raised by the present context in biology and medicine and, in parallel, in bioinformatics. Indeed, these domains are witnessing an actual revolution in the acquisition of molecular data and thus facing a flood of various types of omics data. The ultimate goal is to benefit from the diversity and complementarity of these omics data (data on DNA methylation, copy number variations, polymorphism, etc.) by analyzing them simultaneously. However, multi-omics data integration is only one facet, as we also face an outburst of biocomputational approaches meant to deal with this unprecedented variety and quantity of data, and the choice of a method or of the optimal parameters is generally challenging. In this paper, both simultaneous integration of multiple omics and of various methods are tackled in an innovative manner through an original integration strategy based on consensus.

More specifically, in this work, we address the cancer subtyping problem from a personalized medicine-related perspective, which is gaining increasing attention. To treat patients according to their disease profile, one should be able to distinguish between disease subtypes. These disease subtypes can be predicted from omics data (traditionally gene expression but also methylation, miRNA, etc.) by performing patient clustering (hierarchical clustering, density-based clustering, distribution-based clustering, etc.). Our novel graph-based multi-integration method can fuse multiple input clustering results (obtained with existing clustering methods on diverse omics datasets) into one consensus clustering, regardless of the number of input clusters, number of objects clustered, omics and methods used to generate the input clusterings.

To compute a consensus clustering, our method, implemented in a tool called ClustOmics, uses an intuitive strategy based on evidence accumulation. The evidence accumulation counts (i.e., the number of supports on the integration edges) make the consensus clustering results easier to interpret, as they provide insight into the extent to which the consensus clustering can be considered multi-source (issued from multiple omics) and to the overall agreement of input partitions.

The original EAC strategy as proposed by Fred and Jain [16] uses input partitions obtained by running the K-means algorithm multiple times ($\approx 200$) with random initialization of cluster centroids. From these partition results, a co-occurrence matrix is computed, and a minimum spanning tree algorithm is applied to find consensus clusters, by cutting weak links between objects at a threshold *t* defined by the user. The authors recommend that clusterings obtained for several values of *t* should be analyzed. In ClustOmics, we developed a weighted modularization optimization strategy to automatically select the best filtering threshold. Additionally, rather than generating input clusterings from running the same algorithm multiple times as proposed by Fred and Jain, here, we benefit from using various clustering strategies, each searching for different patterns and giving different insights to the data. This approach also allows the use of algorithms that are specialized for one omics type. Moreover, by taking as input a high number of clusterings obtained with a same tool with varying parameters, the convergence of the consensus clustering, especially in a single-omics context, can be improved, and this improvement can also be achieved by ClustOmics by giving the appropriate input clusterings.

Though our method does not formally weight input datasets (e.g., according to their level of confidence), one can artificially enhance the impact of one or several omics sources by providing supplementary single-omics input clusterings. In the same way, when dealing with missing data, patients measured with all omics are more likely to accumulate supports and therefore more likely to cluster together. In a context of multi-source integration, favoring individuals with the least quantity of missing data makes it possible to highlight the predictions supported by several data sources, which is the desired behavior. In a context of single-source integration, the same set of objects is usually used in all input clusterings (apart from a few specificities of the input clustering tools used).

TCGA real datasets from three different omics and ten cancer types were analyzed with respect to two integration scenarios: (1) fusing multi-omics clusterings obtained with existing integrative clustering tools and (2) fusing omics-specific input clusterings. In both cases, ClustOmics succeeded in computing high-quality multi-omics consensus clusterings, with clusters showing different survival curves and enriched for clinical labels of interest, coherent with what could be found in the cancer literature. Moreover, the results indicate that ClustOmics is robust to heterogeneous input clustering qualities (reconciling and smoothing the disparities of partition) and in comparison with a state-of-the-art consensus-based integration method, COCA.

The overall results show that the rMKL tool outperformed the other tools when considering the survival and clinical label enrichment metrics. However, our method is not meant to compete with existing single or integrative omics clustering methods, as it implements a more generic strategy. While “classical” tools take raw omics data as input, ClustOmics starts from classification results, thus allowing fusing any type of data, as the classification results may correspond to clustering results (obtained from the analysis of one or several omics), to biological annotations, to clinical data, etc.

In contrast, ClustOmics aims at capitalizing on the preliminary input predictions to increase their robustness by taking advantage of accumulating evidence to reveal sharper patterns in the data. This selection of robust patterns across input partitions renders ClustOmics stable when facing heterogeneous input clusterings and is particularly useful when no gold-standard metric is available to assess the quality of the results. Hence, with a sufficient number of input clusterings, no prior analysis of the input is needed, given that low-quality clusterings, likely to add noise to the integration graph, play a smaller part in the evidence accumulation. Omics for which the separation of samples is clear-cut will drive the consensus clustering, while omics that do not show interesting patterns across samples will be faded via the integration graph filtering step. For the same reason, it is important to highlight that as long as the signals in the available omics are strong, ClustOmics is able to cluster samples that do not appear in all omics datasets, making use of available data and addressing the issue of partial data.

Finally, though presented in a disease subtyping context, one should grasp that our method is not limited to this application case. ClustOmics is generic and adaptable to a wide range of biological questions, as one can use any kind of partitioning of the data, including clinical labels, groups of genes of interest, etc., as an input clustering. A major strength of ClustOmics resides in its exploratory aspect, resulting both from a flexible intrinsic model that gives the user complete power on the integration scenario to investigate and from the use of the graph-oriented database Neo4j. All input data and metadata are stored in this kind of database, which may easily be queried and visualized by a nonspecialist with the Neo4j browser.

## Conclusion

Facing the diversity and heterogeneity of omics data and clustering strategies, one might want to make profit from all available data to compute a consensus clustering. ClustOmics is able to fuse any set of input clusterings into one robust consensus, which can easily be interpreted based on the number of supports evidence accumulation scores. ClustOmics can be adapted to answer a wide range of biological questions. The use of integration scenarios allows users to explore various integration strategies, by adding or discarding data sources and/or clustering methods.

## Methods

In this section, we detail the strategy implemented in ClustOmics. We then describe the datasets and the metrics that were used to evaluate our new method. For the sake of simplicity as, in this paper, ClustOmics was applied in the context of cancer subtyping, we will further refer to objects of interest as *Patients*. However, ClustOmics can be applied to different biological entities such as genes or cells.

### ClustOmics integration strategy

**Fig. 7:**
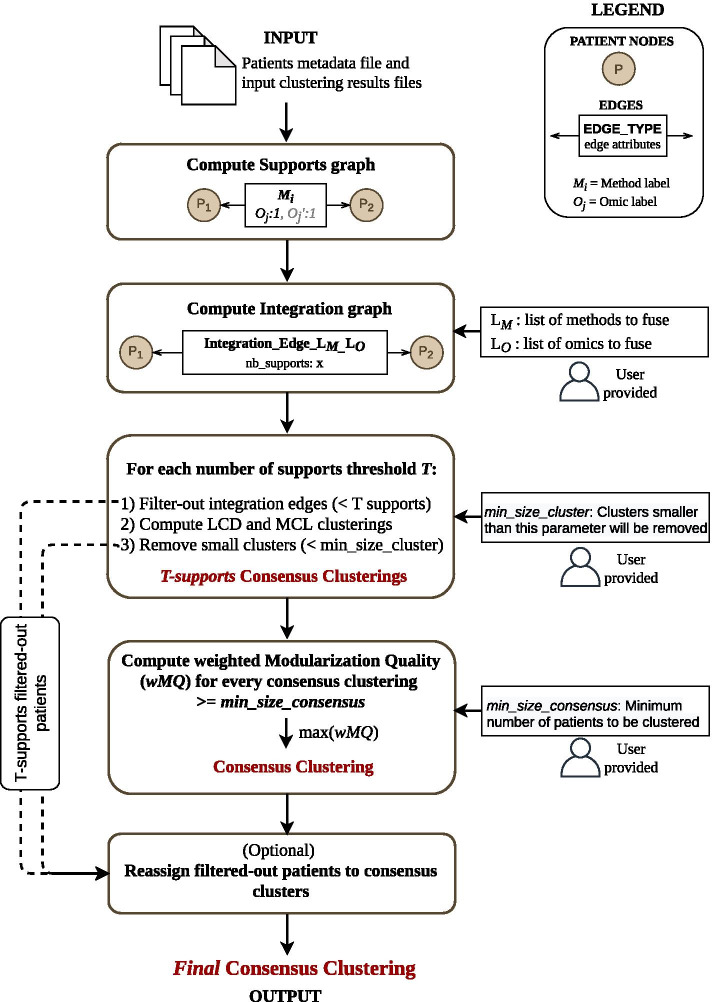
An overview of the strategy implemented in ClustOmics

The ClustOmics integration strategy, depicted in Fig. 7, starts from a set of input clusterings generated with various clustering methods and/or from different omics sources.

**Fig. 8:**
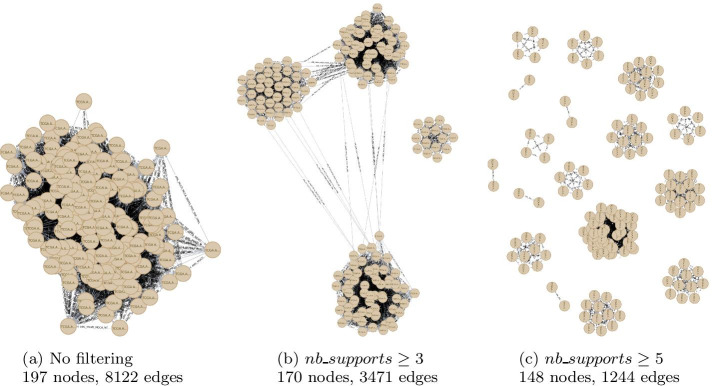
An integration graph filtered with increasing threshold values: 1, 3, and 5 (the maximum number of supports for an integration edge being 5 in this example). Screenshots from the Neo4j browser for graph visualization

First, from a patient metadata file and from available input clusterings, a support graph (SG) is instantiated. In this graph, each *Patient (P)* corresponds to a node and shares a *support edge* with another patient when classified in the same cluster (co-clustered) in at least one input clustering. One support edge relates to one input clustering tool, and each support edge displays one or multiple attributes to indicate the omics sources supporting the co-clustering of the patients.

Next, given an integration scenario, meaning a list of omics and methods to integrate, the corresponding *integration graph (IG)* is computed. Then, the integration graph is filtered and clustered to produce a consensus clustering according to the given integration scenario.

Below, we detail the integration graph computation, filtering and clustering steps, resulting in a ClustOmics consensus clustering.

#### Compute the integration graph (IG)

Given an *Integration Scenario* (defined by a set of input clusterings), ClustOmics exploits the information on the support edges to compute the so-called *number of supports*, by counting the considered input clusterings sustaining the association of patients. The numbers of supports are reported on the *Integration Edges* and so, for a given integration scenario, a pair of patients may share at most one integration edge. In this way, heterogeneous data is aggregated into co-occurrence counts that are used as a similarity measure to perform evidence accumulation clustering (EAC) [16].

#### Filter and cluster the *integration graph*

Integration graphs are generally densely connected, as each pair of nodes may have been clustered together at least once over the set of omics and methods. However, as integration edges are weighted with the number of supports agreeing on the corresponding associations, the most robust integration edges can be distinguished from predictions that are not consistent across omics and methods. Hence, ClustOmics filters the graph according to the number of supports by removing non-consistent integration edges. The goal is to obtain a filtered graph foreshadowing *natural* clusters that correspond to a consensus. The choice of a threshold to filter the integration edges is therefore determinant.

Figure 8 depicts the impact of an increasing number of support-filtering thresholds on the internal structure of the integration graph. One can observe that two issues arise from this filtering process:

- First, increasing the threshold generates smaller graphs. Indeed, pairs of nodes that do not share any integration edge with a sufficient number of supports are removed, leading to a partial classification of the input set of patients.
- The second issue is the loss of structure in the filtered integration graph: when filtering at a high threshold, the resulting graph may become too sparse to be considered informative, like the graph in Fig. 8c with numerous small connected components.

Therefore, producing a relevant classification requires finding the best compromise for the support threshold. For this effort, ClustOmics tests all possible configurations by iteratively filtering the integration graph with increasing support thresholds. At each iteration, ClustOmics uses state-of-the-art graph clustering methods (see the subsection below) to compute consensus clusterings for the corresponding filtered integration graph.

The resulting consensus clusterings should be analyzed with respect to the number of supports used to filter the graph prior to clustering. This number of supports indicates the level of agreement between the input clusterings and gives insight on the extent to which the resulting consensus clustering can be considered as being truly multi-omics.

Moreover, to deal with the two issues described above and to keep merely informative results, each integration graph consensus clustering result goes through an additional filtering step with the two following parameters:

- the *min_size_cluster* parameter indicates the minimum accepted size for a cluster that is part of the consensus clustering. Clusters with less than *min_size_cluster* nodes are removed from the analysis.
- the *min_size_consensus* parameter indicates to what extent ClustOmics is allowed to discard nodes, i.e., patients. ClustOmics will further consider only consensus clustering results having at least *min_size_consensus* nodes.

Finally, a quality metric, i.e., the weighted MQ index, is computed for all consensus clusterings that passed the filtering steps.

Optionally, one may want to reconsider the individuals that were discarded during the filtering steps (either when filtering-out integration edges or small clusters) and analyze them with respect to the consensus clusters. With this in mind, ClustOmics is able to reassign filtered-out individuals with respect to the mean number of supports shared with patients from consensus clusters, though such additional predictions do not necessarily meet the threshold with which the consensus clusters were originally computed.

Below, we give insights on the graph clustering algorithms that are used to compute the consensus clusters, as well as on the quality metric employed for the identification of a robust consensus clustering, the *weighted modularization quality*.

#### Graph clustering algorithms

ClustOmics filters the integration graph for each possible threshold on the number of supports and, for each filtered graph, ClustOmics computes two consensus clusterings with two state-of-the-art, complementary graph clustering algorithms: the Louvain community detection (LCD) algorithm, based on modularity optimization [30], and the Markov clustering (MCL) algorithm, based on the simulation of stochastic flow in graphs [31]. MCL and LCD are unsupervised clustering algorithms and do not require the number of clusters to be estimated in advance.

Modularity optimization is one of the most popular strategies in graph clustering algorithms [32], while MCL/MCL-based methods have proven highly efficient in various biological network analyses (protein-protein interaction networks [33, 34], protein complex identification [35], detection of protein families [36]). Moreover, modularity optimization algorithms have been shown to present a resolution issue [37, 38]: a tendency to fuse small clusters (even for those that are well defined and have few interconnections), thus favoring the formation of larger clusters than those computed by MCL [39]. Small clusters predicted by MCL can be an issue in the ClustOmics case, as it removes clusters smaller than the user-defined *min_size_cluster* parameter, considering them to be non-informative.

#### Selection of the best consensus clustering based on the weighted MQ index

The *modularization quality* (*MQ*) was first defined by Mancoridis et al. in the context of software engineering [40]. Compared to the popular *modularity* measure [41] optimized in graph clustering algorithms, which compares the distribution of edges with respect to a random graph with the same number of vertices and edges as the original graph, the *modularization quality* (*MQ*) evaluates the quality of a clustering as the difference between internal and external connectivity ratios; that is, the ratio between the number of connections observed within a given cluster and between two given clusters, and the maximum possible number of such edges. An optimal clustering for this measure should maximize the intraconnectivity ratio (every two nodes belonging to the same cluster share an edge) and minimize the interconnectivity ratio (nodes classified in different clusters do not share edges). Indeed, in the context of consensus clustering based on evidence accumulation, it makes more sense to compare the distribution of the edges in the integration graph to the case where all nodes would have been partitioned in the same optimal way in all input clusterings, i.e., a graph where all intracluster nodes are connected, and all intercluster nodes are disconnected.

Moreover, we adapted the original *MQ* index for weighted undirected graphs with no self-loops (in our case, a *Patient* node cannot share an integration edge with itself). We denote this adaptation of the modularization quality as the *weighted modularization quality* (*wMQ*).

Let $$G=(V, E)$$ be a graph where *V* denotes the set of nodes and *E* the set of edges of *G*. Let $$C=(C_1,$$...$$, C_k)$$ be a consensus clustering with *K* clusters and $$|C_i|$$ the number of nodes classified in cluster $$C_i$$. Let us also note *w*(*e*) the weight of a given edge, $$W(e_{ii})$$ the sum of weights of the edges internal to $$C_i$$ cluster (connecting vertices from $$C_i$$), $$W(e_{ij})$$ the sum of weights between clusters $$C_i$$ and $$C_j$$ (connecting a vertex from $$C_i$$ to a vertex from $$C_j$$) and $$max(w_{IG})$$ the maximum possible weight on the edges of the given integration graph (the maximum possible number of supports, also corresponding to the number of input clusterings being fused). We therefore define the *wMQ* index computed for a consensus clustering *C* obtained on the integration graph *IG* as:

$$\begin{aligned} wMQ(IG,C) = \frac{1}{k \times max(w_{IG})} \sum _{i=1}^k \left( \frac{2 \times W(e_{ii})}{|C_i| \times (|C_i| - 1)} - \frac{1}{k-1} \sum _{j\ne i} \frac{W(e_{ij})}{|C_i| \times |C_j|} \right) \end{aligned}$$

The first term of the sum corresponds to the weighted internal connectivity ratio for a cluster $C_i$. Indeed, the sum of the internal edges weights $W(e_{ii})$ is adjusted with the maximum possible value of the sum of the edges linking a set of $|C_i|$ nodes, which would be reached if all $C_i$ nodes were connected with $max(w_{IG})$ weighted edges. Note that for an undirected and no self-loop graph, the maximum number of edges in a subgraph of $|C_i|$ nodes is $\genfrac(){0.0pt}0{|C_i|}{2}$. Similarly, the second term of the sum represents the weighted external connectivity ratio of a cluster $C_i$, given by the sum of the weights of the edges linking a node from cluster $C_i$ to a node belonging to a cluster $C_j$ ($\ne C_i$).

The *wMQ* values range from $-1$ to 1, where a *wMQ* of $-1$ corresponds to the case where there is no intracluster edge and all intercluster pairs of vertices are connected with edges of weight $max(w_{IG})$. A *wMQ* of 1 corresponds to the case where no intercluster vertices are connected, and all pairs of intracluster vertices are connected with edges of weight $max(w_{IG})$. A high-standard consensus clustering should maximize this index.

ClustOmics computes the *wMQ* for the LCD and MCL consensus clusterings obtained with various numbers of support-thresholds and having passed the filtering steps, and it returns the clustering that maximizes this quality measure.

### Datasets and tools used for computing input clusterings

We used ClustOmics to predict cancer subtypes from gene expression, microRNA expression and DNA methylation datasets available in *The Cancer Genome Atlas* (TCGA) [42]. Our case-study is based on the same datasets as in *Rappoport and Shamir*’s review on multi-omics clustering methods [1]. The data cover ten cancer types: leukemia (AML), breast (BIC), colon (COAD), glioblastoma (GBM), kidney (KIRC), liver (LIHC), lung (LUSC), ovarian (OV), sarcoma (SARC) and skin (SKCM). For each cancer type, from 197 and up to 1098 patients were measured for at least one of the three omics (expression, miRNA and methylation), of which 170 to 621 patients were measured for all three. More details on missing data per cancer type are given in Table 5.

**Table 5:** Number of patients measured per omics for each cancer type. Total: Number of patients measured for at least one omics (of which those having been measured for the three omics); proportion of partial data; Exp+Met: Patients measured for expression and methylation only; Exp+miRNA: Patients measured for expression and miRNA only; Met+miRNA: Patients measured for methylation and miRNA only; Exp: Patients measured for expression only; Met: Patients measured for methylation only; miRNA: Patients measured for miRNA only

Cancer	Total (multi-omic)	% partial data	Exp+ Met	Exp+ miRNA	Met+ miRNA	Exp	Met	miRNA
AML	197 (170)	13.71	0	3	15	0	9	0
BIC	1096 (621)	43.34	159	132	2	181	1	0
COAD	303 (220)	27.39	57	0	0	8	18	0
GBM	578 (274)	52.60	4	245	3	5	4	43
KIRC	534 (183)	65.73	135	71	0	144	1	0
LIHC	377 (367)	2.65	4	0	5	0	1	0
LUSC	501 (341)	31.94	29	1	0	130	0	0
OV	591 (287)	51.44	7	0	166	9	122	0
SARC	261 (257)	1.53	2	0	2	0	0	0
SKCM	368 (351)	4.62	16	1	0	0	0	0

To generate input clusterings to be fused by ClustOmics, we used five state-of-the-art integrative clustering tools, summarized in Table 6: PINS [6], SNF [7], NEMO [8], rMKL [9] and MultiCCA [10]. The first four tools can be used in a single-omics context as well as in a multi-omics context, for which they were all designed. Though NEMO is the only tool that can handle partial data, for comparability purposes, this functionality was not used in the analyses we conducted. Each tool has been run with default parameters and based on recommendations of the authors. For the single-to-multi scenario, we also computed single-omics input clusterings with K-means clustering [19], for which the optimal number of clusters *K* was determined using the Silhouette index [24], with values of *K* ranging from 2 to 20.

**Table 6:** Methods used to compute input clusterings

Software	Multi-omic context	Single-omic context
PINS [6]	Yes	Yes
SNF [7]	Yes	Yes
NEMO [8]	Yes	Yes
rMKL [9]	Yes	Yes
MultiCCA [10]	Yes	No
k-means [19]	No	Yes

### Computation of COCA consensus clusterings

To assess the performance of ClustOmics with respect to a state-of-the-art integration method based on consensus clustering, we applied cluster-of-clusters analysis (COCA) [5] on each integration scenario and from the same set of input clusterings as for ClustOmics. COCA had already been applied to cancer-subtyping in a multi-omics context [43, 44].

COCA is an integrative clustering tool based on the consensus clustering (CC) algorithm introduced by Monti et al. [45]. The CC algorithm implements a resampling- and co-occurrence-based strategy to assess the stability of clusters when analyzing a single dataset. By resampling a single dataset multiple times and applying a clustering algorithm on each perturbed dataset, and from the co-occurrences counts of samples in clusters, a consensus matrix is computed and used as a similarity matrix to compute a final consensus clustering. COCA was run using default parameters, under the same integration scenarios as in ClustOmics, and using the same set of input clusterings.

### Clustering pairwise similarity metric

To evaluate the similarity of ClustOmics and COCA consensus clusterings with respect to their inputs or each other, we used the adjusted Rand index (ARI) [20], a measure of similarity between two data clusterings. While the ARI has been used to evaluate the quality of classifications compared to ground-truth data, here, we use it to compare the similarity of various clusterings, without considering any quality aspect.

### Biological metrics

To explore the biological relevance of input clusterings and consensus clustering results, we computed the *overall survival rate* of patients. As cancer acuteness is proved to be related to its molecular subtype [46–48], we further investigated whether it was significantly different across clusters using the exact log-rank test for more than two groups, introduced in [49]. For each clustering, the *p* value of the log-rank test was computed using 100, 000 random permutations of the data.

In addition, we performed an analysis of *clinical labels enrichment* in clusters, using 32 labels available from TCGA metadata (see Table 7). The idea is that patients affected by the same cancer subtype should also share, to a certain extent, the same clinical characteristics. The abundance of clinical labels in clusters and their statistical over-representation provide information on the biological robustness of clusterings. To perform this analysis, we used pancancer (e.g., age at diagnosis or pathologic stage of cancer) and cancer-specific clinical labels for each cancer type (e.g., presence of colon polyps for colon cancer, or smoking history for lung cancer). Clinical labels that were absent for more than half of the patients were removed from the analysis. We used the $\chi ^2$ test for independence for discrete parameters and the Kruskal-Wallis test for numeric parameters to assess the enrichment of the clinical labels in a cluster. To increase the robustness of the results, we applied a bootstrapping strategy, computing the test on randomly permuted data to derive an empirical *p* value (100, 000 permutations).

**Table 7:** Pancancer and cancer-specific labels used for clinical label enrichment analysis. Pathological M, N and T labels refer to the TNM staging system, which describes the anatomical extent of tumor cancers [25]

Pan-cancer	Age at initial pathologic diagnosis, Gender, Pathologic M, Pathologic N, Pathologic T, Pathologic stage, Histological type, New neoplasm event type, Neoplasm histologic grade
AML	CALGB cytogenetics risk category, FAB morphology code
BIC	PAM50Call RNAseq, Estrogen receptor status, Progesterone receptor status, ER level cell percentage category, PR level cell percentage category
COAD	Presence of colon polyps, History of colon polyps
GBM	Prior glioma
KIRC	Hemoglobin result, Platelet qualitative result, Serum calcium result, White cell count result
LIHC	Adjacent hepatic tissue inflammation extent type, Albumin result specified value, Creatinine value, Fetoprotein outcome value, Fibrosis ishak score
LUSC	Tobacco smoking history, Number pack years smoked
OV	No supplementary clinical label
SARC	No supplementary clinical label
SKCM	Melanoma Clark level value, Melanoma ulceration indicator

One must keep in mind that molecular data do not always explain survival or clinical differences between groups of samples. Therefore, in the discussion of the results, we consider survival and clinical analysis as ways to interpret patterns captured by the various clustering results and do not favor one metric over the other.

Finally, to evaluate differentially expressed genes across consensus clusters generated using the StoM scenario on the BIC study case, we applied the Kruskal-Wallis test on each gene available from the BIC expression dataset. The *p* values were adjusted to control the false discovery rate (FDR) [50], filtered with a 0.001 significance threshold, and top 1000 most significant genes were retained for further analysis. Using hierarchical clustering [51], we clustered the top gene list and investigated clusters for enriched Gene Ontology [52] biological process terms with Cluster Profiler [53]. FDR-adjusted *p* values were filtered with a 0.05 cutoff.

### Implementation

ClustOmics is implemented based on the Neo4j graph database management system and uses APOC and Graph Data Science Neo4j libraries. Queries on the graph database are performed in Cypher, Neo4j’s graph query language, and are encapsulated in Python scripts. To facilitate its use, ClustOmics can be run through the Snakemake workflow management system.

ClustOmics was tested on a desktop-computer with an Intel Xeon processor (2.70 GHz, 62 GB of RAM) running on Ubuntu 18.04. For the TCGA real datasets that it was applied to, the ClustOmics runtimes range from a few minutes for small datasets (AML, multi-to-multi scenario) up to 2 h for the largest dataset (BIC, single-to-multi scenario), with most of the computation time being consumed for the construction of the integration graph. With the graph stored in a Neo4j database, this step is only to be performed once for each integration scenario, and parameters for graph filtering can be further set and tuned without recomputing the graph.

The ClustOmics source code, released under MIT license, and the results obtained on the ten cancer types with the two integration scenarios described in this paper are available on GitHub: https://github.com/galadrielbriere/ClustOmics.

## Supplementary Information

**Additional file 1**. Supplementary Figures 1 to 4.

## Data Availability

The datasets analysed in the study were obtained from Ron Shamir’s lab and are available at http://acgt.cs.tau.ac.il/multi_omic_benchmark/download.html. ClustOmics’ source code, released under MIT licence, as well as the results obtained on TCGA cancer data are available on Github: https://github.com/galadrielbriere/ClustOmics.

## Abbreviations

AML: Acute myeloid leukemia
ARI: Adjusted Rand index
BIC: Breast invasive carcinoma
CALGB: Cancer and leukemia group B
COAD: Colon adenocarcinoma
EAC: Evidence accumulation clustering
ER: Estrogen receptor
FAB: French–American–British (morphology code)
FDR: False discovery rate
GBM: Glioblastoma
KIRC: Kidney renal clear cell carcinoma
LCD: Louvain community detection
LIHC: Liver hepatocellular carcinoma
LUSC: Lung squamous cell carcinoma
MCL: Markov clustering
MtoM: Multi-to-multi (integration scenario)
MQ: Modularization quality
OV: Ovarian carcinoma
PR: Progesterone receptor
SARC: Sarcoma
SKCM: Skin cutaneous melanoma
StoM: Single-to-multi (integration scenario)
TCGA: The cancer genome Atlas
wMQ: Weighted modularization quality
